# Implementation of a three-tiered approach to identify and characterize anti-drug antibodies raised against HIV-specific broadly neutralizing antibodies

**DOI:** 10.1016/j.jim.2020.112764

**Published:** 2020-04

**Authors:** Pranay Bharadwaj, Cassidy Riekofski, Shu Lin, Michael S. Seaman, David A. Garber, David Montefiori, Marcella Sarzotti-Kelsoe, Margaret E. Ackerman, Joshua A. Weiner

**Affiliations:** a Department of Microbiology and Immunology, Geisel School of Medicine at Dartmouth, Hanover, NH, USA; b Thayer School of Engineering, Dartmouth College, Hanover, NH, USA; c Center for Virology and Vaccine Research, Beth Israel Deaconess Medical Center, Boston, MA, USA; d Division of HIV/AIDS Prevention, Centers for Disease Control and Prevention, Atlanta, GA, USA; e Department of Surgery, Duke University Medical Center, Durham, North Carolina, USA; f Department of Immunology, Duke University Medical Center, Durham, North Carolina, USA

**Keywords:** Anti-drug antibody, HIV, Broadly neutralizing antibody, Safety

## Abstract

The ability to detect, quantify, and interrogate the properties of immune responses raised against biological therapeutics is not only important to our understanding of these molecules, but also to their success in the clinic. A tiered assay approach to identify the presence, specificity, and titer of anti-drug antibody (ADA) responses has been adopted as a gold standard by industry leaders, the FDA, and the EMA. In order to support pre-clinical and clinical trials, these assays must be standardized, and their performance sufficiently characterized to ensure the accuracy and reproducibility of results under relevant testing conditions. Here we present implementation of electrochemiluminiscence assays that fit into the tiered paradigm of ADA testing for five HIV broadly neutralizing antibodies (3BNC117, 3BNC117-LS, 10–1074, PGT121, and PGDM1400) in compliance with Good Clinical Laboratory practices. Assay sensitivities and matrix effects were evaluated and used to inform the development of positivity cut points. Once cut points were established, assay precision, specificity, free-drug tolerance, and robustness were defined. In all cases, assay characteristics met or surpassed recommendations set forth by the FDA. To further evaluate the performance of these assays and the tiered approach, samples from non-human primates that had received a subset of the five therapeutics were evaluated. In sum, this study reports qualification of a set of ADA assays available to the scientific community as pre-clinical and clinical trials of broadly HIV-neutralizing antibodies proceed, and a framework that is easily adapted as new drug products are advanced in the clinic.

## 1 Introduction

In the 30 years since the identification of broadly neutralizing antibodies (bnAbs) to HIV-1, questions have been raised about the feasibility of their use for prevention and treatment via passive administration (Burton et al., 1991; Trkola et al., 2005; Mascola, 2002; Shingai et al., 2014; Mascola et al., 1999; Cavacini et al., 1998; Armbruster et al., 2002; Shibata et al., 1999; Vittecoq et al., 1992; Walker et al., 2011; Sok et al., 2014; Scheid et al., 2011; Mouquet et al., 2012). In contrast to traditional anti-retroviral therapies (ART), bnAbs could remain in circulation longer, reducing the frequency of dosing from daily to perhaps monthly or even biannually. Similarly to ART, bnAbs could be used both prophylactically to prevent infection in high risk populations and as treatment, suppressing viral loads in infected individuals (Ferrari et al., 2018; Margolis et al., 2017; Cohen and Caskey, 2018). Currently, several groups are developing clinical programs to test novel bnAbs or combinations thereof in both uninfected and HIV positive participants. Already, a number of bnAbs have been shown to be well tolerated by individuals (Caskey et al., 2015; Caskey et al., 2017; Caskey et al., 2019). These include those tested in treatment regimens that involve multiple doses (Caskey et al., 2019; Bar-On et al., 2018; Huang et al., 2017; Ledgerwood et al., 2015; Crowell et al., 2019; Mendoza et al., 2018). The Antibody Mediated Prevention (AMP) trials are currently underway to assess the capacity for the bnAb VRC01 to prevent HIV infection in approximately 4300 high risk individuals in the U.S., South America, and Africa. Furthermore, in subjects that are viremic, these interventions can be effective in reducing viral load and/or maintaining viral suppression during analytical treatment interruption. While there are many advantages to using antibodies in HIV prevention and therapy, there are also risks. As compared to small molecular interventions such as ART, these risks can differ for biologics such as antibodies, and include potential immunogenicity, or the propensity to induce an adaptive immune response against the antibody drug.

All biological drug products are likely to induce some level of anti-drug antibody (ADA) which may be harmless, reduce drug efficacy, reduce drug half-life, or be life threatening. The FDA (U.S. Food and Drug Administration) and EMA (European Medicines Agency) both recommend and provide guidance regarding immunogenicity testing for therapeutic protein products during early and late stage clinical trials to understand the prevalence and impact of ADAs for a given drug (“Immunogenicity Assessment of Biotechnology-Derived Therapeutic Proteins,” 2017; Guidance for Industry - Immunogenicity Assessment for Therapeutic Protein Products, 2014; “Immunogenicity Testing of Therapeutic Protein Products — Developing and Validating Assays for Anti-Drug Antibody Detection,” 2019). Among the most popular ADA detection methods is the electrochemiluminescence-based bridging assay, which provides robust and sensitive measurements (Song et al., 2016). In this assay, drug product is aliquoted and conjugated with either biotin or a Sulfo-Tag label. Biotinylated and Sulfo-Tagged drug are then mixed with serum and if ADAs are present, they can generate ternary complexes by acting as a bridge between labeled forms of the drug. ADA responses are then detected on the Meso Scale Diagnostics ™ (MSD) platform based on the generation of luminescence resulting from proximity of the sulfo-tag to the surface of a streptavidin plate that results from the formation of ternary complexes.

Evaluation and characterization of ADAs represent important aspects of evaluating the safety of biologic drugs such as bnAbs (Koren et al., 2008; Mire-Sluis et al., 2004; Shankar et al., 2014; Shankar et al., 2008). ADA detection and assessment require testing and evaluation of a number of parameters that define an antibody as being reactive and detectable in a given serum sample (“Immunogenicity Assessment of Biotechnology-Derived Therapeutic Proteins,” 2017; Guidance for Industry - Immunogenicity Assessment for Therapeutic Protein Products, 2014; “Immunogenicity Testing of Therapeutic Protein Products — Developing and Validating Assays for Anti-Drug Antibody Detection,” 2019). A tier-based approach allows for a logical analysis of a clinical sample and provides for its categorization (Koren et al., 2008; Mire-Sluis et al., 2004; Shankar et al., 2014). To characterize ADAs, a tiered testing approach is often used to first identify, then further define ADA responses that may arise during treatment (Koren et al., 2008; Mire-Sluis et al., 2004; Shankar et al., 2008). In the first tier, a cut point is used to determine whether a sample is positive in a highly sensitive screening assay. If a sample is Tier 1 positive, a confirmatory assay (Tier 2) is performed. Most commonly, competition with free drug is used to determine if the interaction between the drug and sample components is specific. Upon confirmation of the presence of a specific ADA in a clinical sample, a reactivity profile consisting of titer, isotype, subclass and the ability of the ADA to interfere with the interaction between the therapeutic protein and its target (Seaman et al., 2020a) can be generated in Tier 3 (Wu et al., 2016).

Given rapid expansion in the number of clinical trials for new and modified HIV-specific antibodies, developing a robust and reliable platform for measuring and characterizing ADAs in these programs has become a high priority. With this goal in mind, we have implemented a tiered assay approach to measure ADAs against traditional antibody drugs that is also applicable to other biologics such as multi-specifics. Through ADA assay optimization and qualification for five antibodies (3NC117, 3BNC117-LS, 10–1074, PGT121, and PGDM1400), we present a testing approach that is high throughput, has adequate sensitivity, and can be easily adapted as new therapeutics are advanced to the clinic. During ADA assay development, a positivity cut point, competition threshold, and dilution step size to define response titers can be established for each product according to established guidance (Guidance for Industry - Immunogenicity Assessment for Therapeutic Protein Products, 2014; “Immunogenicity Testing of Therapeutic Protein Products — Developing and Validating Assays for Anti-Drug Antibody Detection,” 2019; Validation of analytical procedures: text and methodology Q2: Validation of analytical procedures: text and methodology Q2(R1), 2005). Subsequent qualification enables investigators to be confident in assay specificity, sensitivity, precision, and robustness, as is essential for proper interpretation of results. Finally, because the ADA binding assays will directly aid investigators in defining efficacy and supporting pharmacovigilance, qualification ensures that the assays operate within the definitions of Good Clinical Laboratory Practice (GCLP) (Sarzotti-Kelsoe et al., 2014; Sarzotti-Kelsoe et al., 2009).

## 2 Materials and methods

### 2.1 Preparation and handling of bnAb drug products

Drug products were provided by the laboratories of Dr. Michel Nussenzweig (Rockefeller University, NY) and Dr. Dan Barouch (Beth Israel Deaconess Medical Center, MA). As needed, bnAb drug products were desalted with a Zeba spin desalting or Amicon centrifugal columns to remove buffers containing amine groups. Antibodies at concentrations of 1–2 mg/mL were covalently conjugated with biotin (EZ-Link™ Sulfo-NHS-LC-Biotin from Thermo Fisher) at a molar ratio of 1:10 and with S-tag (Meso Scale Discovery (MSD) GOLD SULFO-TAG NHS-Ester label) at a molar ratio of 1:12 according to the manufacturers' recommendations. Biotinylation of PGT121 occurred at a challenge ratio of 1:50 as it was noted that additional sensitivity was gained. Briefly, fresh stocks of each S-Tag and biotin were prepared in molecular grade water at a concentration of 1–3 and 10 mM, respectively, then incubated at room temperature for two hours. Excess label in solution was removed after conjugation using either Zeba spin desalting columns or Amicon centrifugal columns. Labeled drugs were stored at 4 °C in either PBS or conjugation storage buffer (MSD R60AC-1).

### 2.2 Specimens

Treatment naïve HIV+ and/or HIV- serum samples were acquired from SeraCare (Milford, MA), The Rockefeller University (New York, NY), and BioreclamationIVT (Westbury, NY). Pooled normal human serum (PNHS) was sourced from Millipore (St. Louis, MO). The study was approved by The Rockefeller University and Dartmouth.

College Institutional Review Boards, and each subject gave written informed consent. Longitudinal plasma samples were obtained from non-human primates (NHP) administered 3BNC117 and/or 10–1074 approved by the appropriate Institutional Animal Care and Use Committee at the Center for Disease Control.

### 2.3 Control reagents

Monoclonal murine anti-idiotypic (anti-id) antibodies for each drug product were provided by investigators for use as positive controls as follows: anti-PGDM1400 (IV1919.265.37, Covance), anti-PGT121 (IV1737.939.26, Covance), anti-3BNC117 (1F1–2E3, Duke Protein Production Facility), and anti-10-1074 (A1-4E11, Duke Protein Production Facility). Rheumatoid Factor (RF) was sourced from Athens Research and Technology.

### 2.4 Assay preparation

Biotinylated and sulfo-tag labeled mAb were mixed at a 1:1 M ratio and diluted in 1% PBSB (PBS with 1% MSD blocker A) to generate a master mix. MSD gold 96 well streptavidin plates (MSD L155A-1) were blocked for at least 30 min on an orbital plate shaker at 600–900 RPM and RT with 150–170 μL 3% PBSB (PBS with 3% MSD blocker A), or alternatively blocked at 4 °C overnight without shaking, prior to washing with 150–200 μL PBST once either using a plate washer (Bio-Tek) or by hand. Residual liquid was removed by flipping the plate and gently tapping.

### 2.5 ADA assay

Specimen incubation plates were prepared by combining 2–73 μL of each sample to be tested with a quantity of ADA master mix to bring the final volume to 75 μL and each drug-conjugate to a final concentration of 0.67 μg/mL (for PGT121, 0.9 μg/mL was the final concentration of each drug-conjugate). Plates were sealed and incubated on an orbital plate shaker at 600–900 RPM for 1–2 h at room temperature (RT). A volume of 50 μL from each well from the specimen incubation plate was transferred to the blocked streptavidin assay plate and put on an orbital plate shaker at 600–900 RPM for 1 h at RT. Following incubation, assay plates were washed with 150–200 μL PBST three times either by hand or using a plate washer, and 150 μL of 0.5-2× MSD Read Buffer T (MSD R927C-1) was added to each well prior to data acquisition (MSD QuickPlex SQ 120, Meso Discovery Workbench Software 3.0). Results are reported as signal to baseline (S/B) ratio values, where plate-specific baseline values were defined by the means signal observed for two or more wells containing only PBSB.

### 2.6 Free drug competition

Addition of unlabeled free drug was used to assess assay specificity in Tier 2. For competition tests, a quantity of unlabeled bnAb equivalent to a 10 μg/mL serum concentration was added to each test specimen. The unlabeled mAb and specimen mixture was incubated on an orbital plate shaker (600–900 rpm) at RT for 1 h prior to addition of the labeled drug master mix and ADA testing as described above.

### 2.7 Statistics and calculations

For the purposes of understanding how much baseline ADA signal is present in a subject population which can then be used to establish positivity thresholds and define sensitivity, a Qualification Cut Point (QCP) was established. The QCP was defined using the approach defined by Shankar et al. (Shankar et al., 2008). Briefly, a set of 99–129 samples from a representative bnAb naïve subject population were evaluated in the screening assay over six replicate runs. Subjects exhibiting signal greater than the third quartile plus 1.5 times the interquartile range (Excel) were deemed outliers and eliminated from cut point analysis. The Shapiro-Wilk test was used to evaluate normality (Graphpad Prism 7.02) and the 95th percentile was calculated using the PERCENTILE function in Excel.

To establish if a clinical sample is ADA positive, a Screening Cut Point was determined from qualification data. When both the means and variances were similar across the six runs, the screening cut point was defined as equal to the QCP. When the means were different (One-way ANOVA, Graphpad Prism 7.02) and the variance of the means of the six runs were similar (Brown-Forsythe, Graphpad Prism 7.02), a floating cut point was defined. The floating cut point calculates a plate-specific correction factor defined as the difference between the average PNHS signal observed during qualification and the QCP, and adds this correction factor to the mean signal observed for PNHS samples tested within the run.

To determine if there were differences in data collected under different assay conditions, *t*-tests or ANOVA tests were performed in GraphPad Prism 7.02. In some cases, such as when determining variability between replicates, % coefficients of variability (CV) were calculated in Excel.

For determination of specificity in Tier 2, percent signal reduction for each sample that did not exceed the QCP was calculated as follows:

$$\%\mathrm{reduction}=\frac{100*\left(S/B\_\text{Untreated}-S/B\_\text{With Drug}\right)}{S/B\_\mathrm{Untreated}}$$

Outlying percent reduction values were eliminated using the ROUT method in GraphPad Prism 7.02 (Q = 1%) and the distribution of remaining values were analyzed from each run using the Shapiro-Wilk normality test (GraphPad Prism 7.02). For normally distributed data, the 99th percentile of % reductions for each run was calculated using the Mean + 2.3263*Standard Deviation, and the average 99th percentile of the two runs was defined as the % Inhibition Threshold. In the case of non-normal distributions, the PERCENTILE function in Excel was used.

To identify a titer step size that would reliably capture differences in ADA levels that were likely to be of biological rather than technical origin, the fold change of individual replicates from the median across six replicates was calculated for any sample tested for which at least one replicate exceeded the QCP. The distribution of fold changes observed was used to estimate the rate at which a given fold change would be expected to result from technical variability.

## 3 Results

### 3.1 Tiered assay approach

Testing to identify and appraise ADA responses is an important aspect of evaluating the safety and efficacy of biologic drugs, albeit one that is made difficult by the need to develop a comprehensive approach to detect and characterize responses to products that have yet to be administered to human subjects, meaning that no samples representing natural human humoral responses are available to serve as positive controls. Nonetheless, even in the absence of human specimens to serve as true biological positives, the FDA, EMA, and industry leaders have established guiding principles that form the basis for developing such tests (“Immunogenicity Assessment of Biotechnology-Derived Therapeutic Proteins,” 2017; Guidance for Industry - Immunogenicity Assessment for Therapeutic Protein Products, 2014; “Immunogenicity Testing of Therapeutic Protein Products — Developing and Validating Assays for Anti-Drug Antibody Detection,” 2019; Koren et al., 2008; Mire-Sluis et al., 2004; Shankar et al., 2014; Shankar et al., 2008). This guidance provides recommendations for development of sufficiently sensitive, specific, and repeatable tests to enable investigators to understand the immunogenicity profile of a given biologic (Table 1). Additional recommendations from the FDA, EMA, and Good Clinical Laboratory Practices (GCLP) guidance documents include the evaluation of assay robustness and performance under relevant testing conditions (Table 2) (“Immunogenicity Assessment of Biotechnology-Derived Therapeutic Proteins,” 2017; Guidance for Industry - Immunogenicity Assessment for Therapeutic Protein Products, 2014; “Immunogenicity Testing of Therapeutic Protein Products — Developing and Validating Assays for Anti-Drug Antibody Detection,” 2019; Sarzotti-Kelsoe et al., 2014; Sarzotti-Kelsoe et al., 2009). Here we report implementation of an approach to define and characterize ADA responses raised against the HIV-specific broadly neutralizing antibodies (bnAbs) 3BNC117, 3BNC117-LS, 10–1074, PGDM1400, and PGT121.

**Table 1:** Qualification parameters, methods, FDA guidance, and derived results. Assay performance and characteristics are reported along with summarized FDA guidance for the five HIV bnAbs tested.

			Derived Assay Criteria				
Qualification parameter	Experimental design	FDA Guidance	10–1074	3BNC117	3BNC117LS	PGDM1400	PGT121
Serum dilution factor	Dilutions of anti-id in multiple concentrations of PNHS were used to estimate matrix effects and identify a suitable serum dilution.	< 1:100	1:12	1:12	1:12	1:6	13:1
Sensitivity	Dilutions of anti-id were run in duplicate. Assay sensitivity (ng/mL of ADA in serum) was determined using the qualification cut point.	< 100 ng/mL	22.9	11.1	22.9	0.293	31.3
Repeatability	QC samples were run by multiple operators over several days. Within plate % CVs were calculated.	< 20%	4.7%	3.3%	5.5%	6.5%	7.5%
Intermediate precision	QC samples were run by multiple operators over several days. Between plate % CVs were calculated.	< 20%	2.1–4.9%	1.9–5.9%	3.1–4.5%	6.0–6.5%	4.8–6.7%

**Table 2:** Assay characteristics, methods, and summarized observations. Assay characteristics are reported along with methods and details for the five HIV bnAbs tested.

	Experimental design		10–1074	3BNC117	3BNC117LS	PGDM1400	PGT121
Cut Point	Treatment-naïve HIV+ and HIV- subjects' serum samples were assayed	Fixed	2.7 S/B	1.7 S/B	2.2 S/B	1.45 S/B	3.0 S/B
		Floating	+0.7 S/B	+0.4 S/B	+0.3 S/B	+0 S/B	+0.7 S/B
Specificity	Serum samples from three different categories of patients (HIV-, HIV+ ART+, and HIV+ ART-) were tested in duplicates, and then retested with additional anti-id spiked-in.		All samples became ADA positive upon anti-id spike in	All samples became ADA positive upon anti-id spike in	All samples became ADA positive upon anti-id spike in	All samples became ADA positive upon anti-id spike in	HIV+ ART+ and HIV- samples all became ADA positive ART- subjects had reduced anti-id induced signal. (5/16 remained below cut point)
	Serum samples were spiked with 3200 ng/mL of an unrelated mAb or 20 IU/mL of rheumatoid factor (RF) and assayed.		2 of 13 samples became positive upon addition of either mAb or RF.	0 of 13 samples became positive.	0 of 12 samples became positive.	0 of 10 samples became positive.	6 of 13 samples became positive upon addition of anti-PGDM1400 All 13 samples became positive upon addition of RF
Drug tolerance	PNHS containing anti-id at concentrations sufficient to result in a positive response call were spiked with the respective mAb over a range of concentrations and assayed in duplicate by two operators. An unrelated mAb was also spiked into samples containing the mAb anti-id.	100 ng/mL anti-id^a^	70 μg/mL	10 μg/mL	30 μg/mL	12.5 μg/mL	1.9 μg/mL
		500 ng/mL anti-id	>140 μg/mL	>60 μg/mL	>60 μg/mL	>40 μg/mL	1.3 μg/mL
		Unrelated mAb	140 μg/mL 3BNC117 did not result in any signal interference	80 μg/mL 10–1074 did not result in any signal interference	60 μg/mL 10–1074 did not result in any signal interference	40 μg/mL PGT121 did not result in any signal interference	20 μg/mL PGDM1400 did not result in any signal interference
Stability	QC samples were subjected to either freeze-thaw cycles, three hours at room temperature, or three days at 4 °C, then compared to samples that were stored at −80 °C by two operators	Stability of the samples was tested through three freeze-thaw cycles, for three hours at room temperature (RT), and for three days at 4 °C.	RT: 3 h 4 °C: 3 days −80 °C: 3 months	RT: 3 h 4 °C: 3 days −80 °C: 3 months	RT: 3 h 4 °C: 3 days −80 °C: 3 months	RT: 3 h 4 °C: 3 days −80 °C: 3 months	RT: 3 h 4 °C: 3 days −80 °C: 7 months

a PGT 121 drug tolerance was established with a spike in of 200 ng/mL anti-Id.

**Fig. 1:**
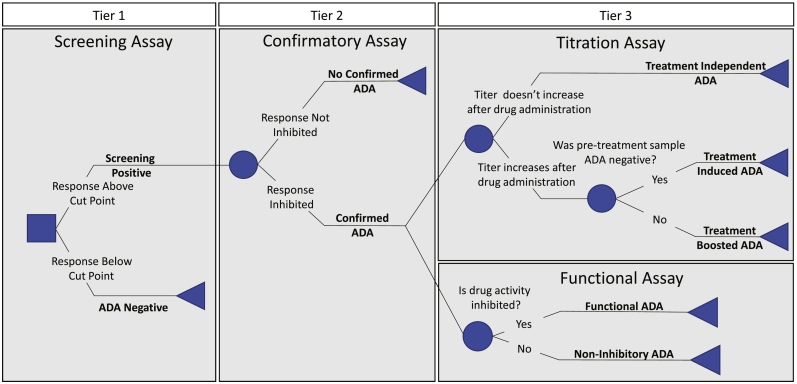
ADA detection using a tiered approach. Tier 1 (left) serves as a high-sensitivity preliminary screen used to define samples as ADA negative or screening assay positive. Tier-2 (center) provides confirmation of the presence of an ADA; in this case, evaluating the specificity of Tier 1 positive responses by determining the ability of excess drug to competitively inhibit the signal observed. Tier 3 (right) consists of assays aimed to characterize confirmed ADA responses. Titrations are performed to define the magnitude of the ADA response as well as whether observed responses were induced, boosted, or independent of drug treatment as defined by longitudinal sampling (top). The ability of the ADA response to impact the biological activity of the bnAb is assessed in a functional assay (bottom), in which a modified HIV neutralization assay may be used to define the ability of the ADA response to inhibit the bnAb's ability to neutralize virus.

Commonly, a three-tiered approach is employed for testing (Fig. 1) (Swanson, 2003; Swanson et al., 2004). Tier 1 is comprised of a screening assay designed to have high sensitivity. Here, the popular “bridging” assay format using electrochemiluminescent detection of complexes of anti-drug antibody with differentially labeled forms of drug is employed. The screening assay uses a cut point designed to pass a minimum of 5% of samples from a representative population of drug naïve subjects forward to a second tier confirmatory assay designed to have high specificity. Samples with signal below the cut point in Tier 1 are defined as ADA negative and are not investigated further. Those exhibiting signal above the cut point are defined as screening assay positive and are then evaluated in the Tier 2 confirmatory assay. Here, the confirmatory assay defines the specificity of the response by determining the extent to which free drug can inhibit the response observed in the bridging assay. Samples exhibiting inhibition greater than the 99th percentile of inhibition observed among samples from drug naïve subjects are defined as possessing a confirmed ADA response (Shankar et al., 2008). Upon confirmation of the presence of ADA in a clinical sample, a reactivity profile consisting of the titer, isotype, subclass, and the interference ability of the ADA with the therapeutic protein and its target, or other characteristics of interest may be generated in Tier 3. Here, ADA responses are characterized to define their magnitude, or titer, and, on the basis of their longitudinal profile, whether these responses were induced, boosted, or observed independently of drug treatment (Shankar et al., 2014). While it is often difficult to develop assays to determine the functional consequences of an ADA response (Gupta et al., 2007), in the case of HIV bnAbs, the ability for ADAs to inhibit the bnAbs' capacity to neutralize virus infectivity can also be measured with relative ease by adaptation of a widely used virus neutralization assay (Seaman et al., 2020b). Overall, the tier-based assessment provides an identification and characterization framework that is easily adapted to diverse bnAbs.

### 3.2 Optimizing the tier 1 screening assay

Using monoclonal anti-idiotypic antibodies raised in mice as positive controls, experiments were conducted for each bnAb to define and optimize testing conditions that met FDA/industry guidance for screening assay sensitivity. This guidance suggests no more than a 100-fold dilution of serum and sufficient sensitivity to detect an anti-idiotypic response of 100 ng/mL or less in serum. Given reliance on monoclonal anti-idiotypes with differing characteristics, testing conditions that satisfied this guidance were somewhat different across the panel of bnAbs; most notably, they varied from testing serum at a dilution of 1:12 for 10–1074 and 3BNC117, to almost neat serum (13:1) for PGT121 (Table 1). Other aspects of optimized assay conditions, such as the extent of drug labeling, concentrations of drug used, ratios and concentrations of labeled drug products, time of incubation, and use of signal to baseline (S/B) ratios as opposed to raw signal intensities were typically consistent across bnAbs (data not shown).

### 3.3 Establishment of the tier 1 screening assay positivity cut point

**Fig. 2:**
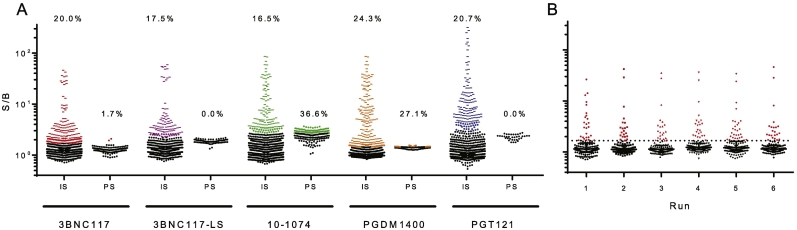
Tier 1 screening assay cut point determination. A. Tier 1 screening assay signal over baseline (S/B) observed for repeated (*N* = 6) measurements of individual serum (IS) samples (*N* ≥ 99) and pooled HIV- human sera (PS) from drug naïve subjects for each of the indicated bnAbs. For each bnAb, data points defined as outliers based on quartile three plus 1.5× the interquartile range are indicated in color. Inset values indicate the rate of Tier 1 screening assay positivity observed among this set of individual and pooled sera from drug naïve subjects. B. S/B responses observed across repeated screening assay runs (N = 6) for 3BNC117 (*N* = 129, each dot represents the mean of technical duplicates). Dotted line represents the screening assay cut point. Data points defined as Tier 1 positive are indicated in color. Bar and whiskers represent median with interquartile range).

The Tier 1 assay provides classification of a test sample as positive or negative in the ADA screening assay. In order to set the threshold defining screening assay positivity, analysis of serum samples from a cohort of 50 or more representative, treatment-naïve subjects was performed in replicate to quantify biological and assay-related noise. For each of the HIV bnAb screening assays, a set of 99 or more serum samples from treatment-naïve HIV+ and HIV- subjects were assayed by at least two different operators on three different days for a total of six assay runs (Supplemental Table 1). Data from these runs were combined (Fig. 2A) and analyzed as recommended by FDA guidance (Guidance for Industry - Immunogenicity Assessment for Therapeutic Protein Products, 2014; “Immunogenicity Testing of Therapeutic Protein Products — Developing and Validating Assays for Anti-Drug Antibody Detection,” 2019) following the procedure established by Shankar et al. (Shankar et al., 2008), which has been widely accepted as a gold standard for cut point determination. Outliers were identified as any datapoints exceeding the third quartile plus 1.5-fold the interquartile range and were eliminated from further consideration in cut point analysis. The Shapiro-Wilk normality test indicated that the remaining data was non-normally distributed for each bnAb, and given these distributions, the 95th percentile was used to define the qualification cut point (QCP). The 95th percentile, reflecting a conservative approach to defining screening assay positivity, is recommended in order to avoid the misclassification of samples as ADA negative. Following established procedure (Shankar et al., 2008), the means of individual runs (Fig. 2B) were compared to each other using one-way ANOVA, and variances were compared using the Brown-Forsythe test to determine the appropriate approach to set the screening cut point (SCP). For all five bnAbs tested, no significant differences were observed between the means or variances between assay runs. As such, it was acceptable to use either a fixed cut point equivalent to the QCP or to use a floating cut point as the screening assay positivity threshold. A floating cut point, which is based on the signal from a given standard, in this case PNHS, which is tested in each plate, can be useful if run to run variability is of concern.

The S/B values defined to signify assay screening assay positivity varied from 1.45 to 3.0 across the panel of bnAbs. Given exclusion of outliers prior to setting the 95th percentile, the screening assay identified 17.5–24.3% of samples as Tier 1 positive. While it is challenging to compare the samples used in the QCP analysis for the different drugs due to different serum percentages used, 99 samples were tested in common across all four of the parent molecules (Supplemental Table 1), and no statistically significant correlations were observed for S/B measures between drugs across common samples, indicating that responses observed among treatment naïve subjects showed specificity for one drug over another, and suggesting the potential biological relevance of this variation. The pooled serum sample (PNHS) employed for this test exhibited a range of S/B response magnitudes, sometimes exceeding the assay cut point. Reproducibility was acceptable (CV < 20%), and the fraction of PNHS replicates that fell above the positivity cut point varied from 0 to 36.6%, consistent with inherent differences in the ability of this serum pool to interact with and bridge the different drug products.

### 3.4 Establishment of screening assay sensitivity and specificity

**Fig. 3:**
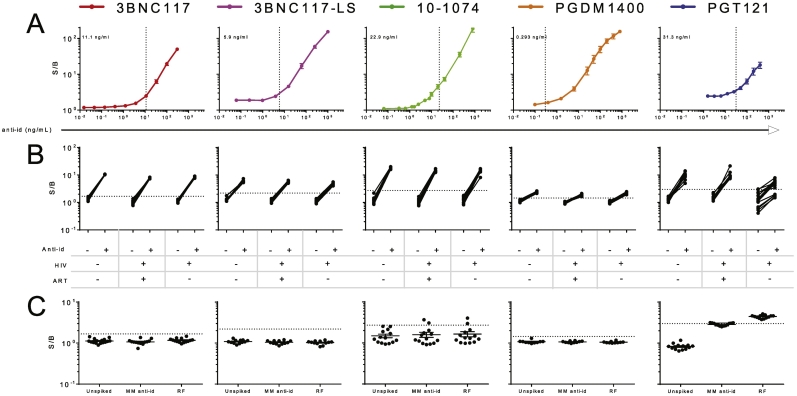
Tier 1 screening assay sensitivity and specificity. A. S/B values observed across titrations of anti-id for each bnAb. Dotted lines and inset values report the calculated sensitivity for each assay's ability to detect its matched monoclonal anti-id. Error bars represent the standard error of the mean. B. S/B responses observed among drug naïve subjects (*N* = 38–43, broken out by infection (HIV +/−) and therapy (ART +/−) status), and with and without an anti-id spike. C. S/B values observed among drug naïve sera samples (*N* = 10–14) when spiked with 3200 ng/mL of a mismatched (MM) anti-id or 20 IU/mL of rheumatoid factor (RF). Dotted lines indicate the screening assay positivity cut point. Bar and whiskers represent mean and standard error of the mean.

While the screening assay cut point is defined only by serum samples from representative subjects, determination of assay sensitivity requires a positive control, commonly either a polyclonal serum sample or a monoclonal anti-idiotypic (anti-id) antibody isolated from a drug-immunized animal. Because these positive controls are presumed to differ in their affinity and epitope specificity, they are also presumed to have different inherent capacities to bind and bridge the drug product in the screening assay; indeed, use of different monoclonal anti-ids can result in different reported sensitivity values for the same bnAb in the same assay (Mire-Sluis et al., 2004). Nonetheless, the FDA has established a target sensitivity of <100 ng/mL, and different assay conditions (serum dilutions) were required to meet this recommendation across the panel of bnAbs. To formally define ADA screening assay sensitivity, anti-ids were spiked into pooled normal human serum, titrated, and measured in a set of six assay runs performed by three different operators on three different days. Assay sensitivity was defined as the mean minimum concentration of anti-id at which a signal above the cut point was observed across repeated assay runs (Fig. 3A). For some bnAbs, signal from pooled normal human sera was higher than the cut point defined by signal observed from samples from individual treatment naïve subjects, and thus was not representative. In these cases where the average PNHS value exceeded the qualification cut point, anti-id was titrated in PBSB instead of PNHS. This approach resulted in similar sensitivities as calculated by interpolation of the linear portion of an anti-id curve titrated in PNHS.

In conjunction with the titrations of anti-id to determine sensitivity, the effect of high concentrations of ADA were also examined. At the upper end of the titration curve, a “hook” or “prozone” effect was observed wherein as more anti-id was added, signal began to decrease (Butch, 2000). This phenomenon is relatively common in immunoassays; here it is presumably the product of having a sufficient excess of ADA so that when labeled products are added to the test sample, it becomes more likely that the ADA will interact with only one of the two differently labeled drugs. The prozone effects observed here were apparent only at very high ADA concentrations, at which only modest signal suppression was observed, such that false negative results due to the prozone effect are not expected at physiologically relevant concentrations. Given that the binding assay is qualitative and the magnitude of a Tier 1 response is not used to interpret the quantity of ADA raised, the prozone effect does not appear likely to impact the results of ADA testing with these products.

To confirm the assays' ability to detect low quantities of ADAs under diverse biological conditions, serum samples from individuals representative of different subject groups anticipated to participate in clinical studies (HIV, HIV+ ART+, and HIV+ ART-) were evaluated in duplicate with and without 100 ng/mL (200 ng/mL for PGT121) of anti-id spiked in (Fig. 3B). Because this concentration of anti-id is above the sensitivity level of the ADA screening assay, each of the spiked samples is expected to be called ADA positive based on exhibiting an S/B greater than the cut point. The anti-id spike-in resulted in Tier 1 positivity for all drugs and all groups, with one exception; 5 of the 16 serum samples from individuals who were HIV infected but not currently on ART did not exceed the assay cut point after the spike in of anti-PGT121, indicating that this screening assay may have reduced sensitivity in this population. In contrast, and to evaluate assay specificity, when samples were spiked with 3200 ng/mL of a mismatched anti-id, the great majority of samples remained screening assay negative (Fig. 3C). To further test the impact of non-idiotype-specific matrix components, such as rheumatoid factor (RF), on screening assay results, samples were spiked with 20 IU/mL of RF, a level associated with adverse clinical prognosis (Araujo et al., 2011; Tatarewicz et al., 2010), and evaluated for screening assay positivity. With the exception of PGT121 and 10–1074, this level of RF did not result in assay positivity. In the PGT121 assay, all samples became positive, while in the 10–1074 assay, 2 of 14 samples became positive (Fig. 3C), indicating that Tier 1 assay positivity can result from matrix components such as high levels of RF for these drug products.

### 3.5 Screening assay precision

**Fig. 4:**
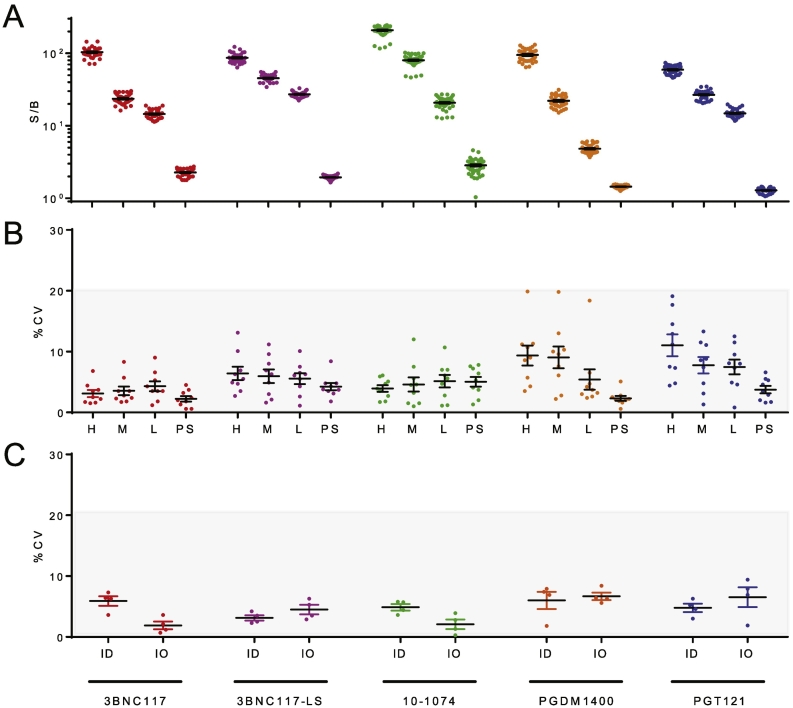
Intra and inter-assay variability. A-B. Intra-assay variability. S/B (A) and percent coefficient of variation (%CV) (B) for technical replicates (*N* = 35–46) of the high (H), medium (M), and low (L) anti-id concentration control samples and pooled sera (PS) are plotted. C. Inter-assay variability as assessed by measuring the coefficient of variation between inter-operator (IO) and inter-day (ID) measurements. Bar and whiskers indicate mean and standard error of the mean. The range of %CVs generally recommended as acceptable for biophysical assays is indicated in gray.

Precision or repeatability is defined by the extent of variability observed between replicate samples that have been treated and assessed as identically as possible (e.g. run at the same time, on the same plate, by the same operator). Four replicates of high (200–3200 ng/mL), medium (50–800 ng/mL), and low (12.5–200 ng/mL) levels of anti-id were spiked into pooled serum. These three positive controls as well as pooled serum alone were assayed by at least two separate operators over at least three days for a total of nine runs, for an assessment of intermediate precision (Fig. 4A). Variability across these replicated runs was assessed by determination of the average coefficient of variation (%CV) observed (Fig. 4B). On average, across the panel of bnAbs, average assay repeatability ranged from 3.3% to 7.5%. Variability tended to be somewhat greater in tests in which higher concentrations of serum were used, such as for PGDM1400 and PGT121, for which 1:6 and 13:1 dilutions of serum, respectively, were employed. No striking differences in variability among samples with different signal magnitudes were noted. Lastly, assay intermediate precision was evaluated and revealed that variability among assays conducted by different operators on the same day (average %CVs ranging from 1.9% to 6.7%) tended to be lower than that observed day to day (average %CVs ranging from 3.1 to 6.0) (Fig. 4C), although all fell within the recommended range.

### 3.6 Free drug tolerance

**Fig. 5:**
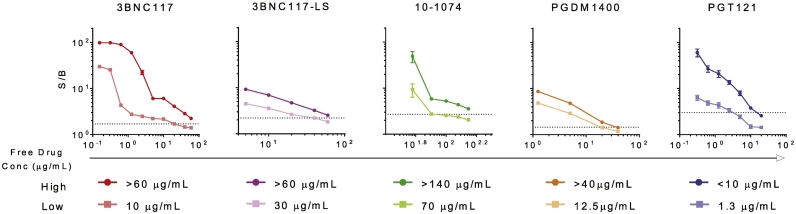
Free drug tolerance. The ability of free drug to reduce S/B signal was evaluated at high (500 ng/mL) and low (100 ng/mL for all bnAbs except PGT121 which was run at 200 ng/mL) levels of anti-id. Dotted lines indicate the screening assay positivity cut point. Bar and whiskers indicate standard error of the mean. Tabulated values indicate the maximum tested concentration of free drug that could be added to high and low levels of anti-id without resulting in a false negative response call.

In treated subjects, particularly at early timepoints, ADA assessments are complicated by the potential for unlabeled drug present in serum to compete with the labeled drug added for testing purposes in the formation of complexes, resulting in signal suppression and the possibility of false negative determinations (Zhong et al., 2017). While the pharmacokinetics of a mAb will vary by route of administration, dosage, and antibody attributes, when VRC01, a mAb with an unmodified Fc was given as a single 20 mg/kg IV dose, the levels of circulating VRC01 in serum dropped to ~10 μg/mL after 8 weeks (Ledgerwood et al., 2015). While the FDA does not have formal drug tolerance recommendations, the level of interference imparted by free drug against a positive control should be defined in order to aid in selection of timepoints and in interpretation of negative results in light of drug pharmacokinetics. Accordingly, free drug was titrated into pooled serum samples containing anti-id at high (500 ng/mL) and low (100–200 ng/mL) levels, and assayed in duplicate by two operators. Assay drug tolerance was defined as the highest concentration of drug tested that did not prevent the detection of anti-id (Fig. 5). Across the set of bnAbs, drug tolerance in detection of low and high levels of anti-id ranged from 1.3 to 70 μg/mL, and 1.3 to >140 μg/mL, respectively. Again, although only representative of the drug tolerance in detection of the specific anti-id used in testing, this measure may serve as a useful parameter to consider in the selection of appropriate timepoints for testing, as well as in understanding the caveats in interpretation of ADA test results.

### 3.7 Establishment of the tier 2 specificity threshold

**Fig. 6:**
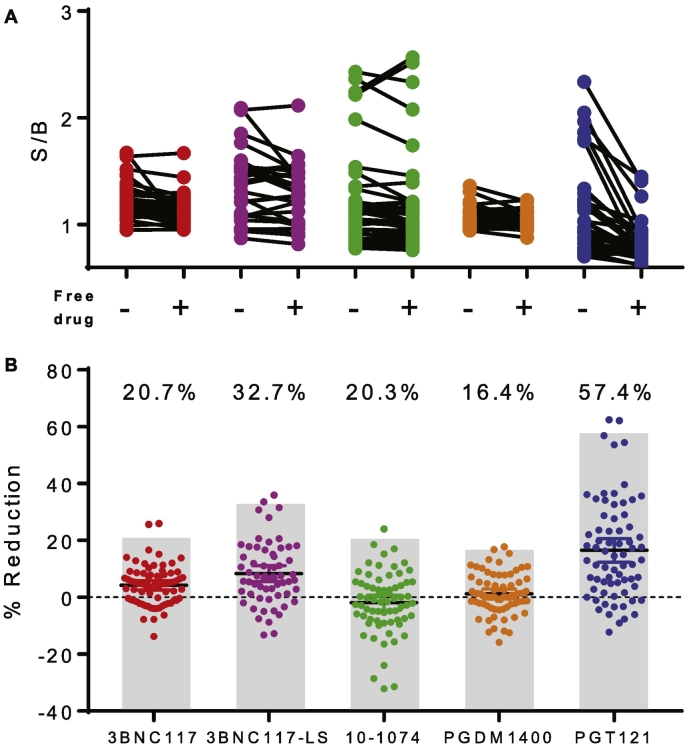
Tier 2 confirmatory assay specificity determination. A. S/B values observed among drug naïve subjects (*N* = 31–37) with and without addition of 10 μg/mL of free drug. B. The percent reduction in S/B resulting from spiking with free drug was calculated for each sample. Gray bars and inset values indicate the threshold defining a specific or Tier 2 positive response, set at the 99th percentile of the observed distribution of degree of signal reduction among samples from drug naïve subjects for each bnAb.

Given the conservative cutoff applied in the Tier 1 screening assay, a considerably stricter threshold is recommended in Tier 2. Here, a specificity test is employed as the confirmatory assay. To this end, the screening assay described above is conducted in the presence of additional free drug product as a competitor in order to confirm ADA responses by defining them as specific or non-specific. Responses are deemed specific by virtue of exhibiting signal suppression exceeding the 99th percentile of reduction observed among samples from drug naïve subjects (Shankar et al., 2008). In order to define this threshold, a set of 40 samples from drug naïve subjects was evaluated with and without a plasma equivalent of 10 μg/mL free unlabeled drug spiked in (Fig. 6A). Following the exclusion of samples that exceeded the QCP without free drug present, percent signal reduction for each sample was calculated. Outlying percent reduction values were eliminated and the remaining distribution of these values was used to define the 99th percentile of signal reduction observed (Fig. 6B). Because the same 40 samples were evaluated for all four of the parent drugs (PGT121, PGDM1400, 3BNC117, and 10–1074), a comparison of the identity of the samples that were deemed Tier 2 positive was performed (Supplemental Table 2) and it was observed that no sample was Tier 2 positive for more than a single drug, again indicating that responses observed among treatment naïve subjects showed specificity for particular drugs. Suitability of these thresholds to demonstrate the specificity of matched but not unmatched anti-ids as true positives was confirmed (data not shown). In sum, of the 52 Tier 1 positive samples, 21 were defined as specific. This observation suggests that the tiers are providing distinct rather than redundant testing information, as intended.

### 3.8 Establishing criteria for titering responses in tier 3

**Fig. 7:**
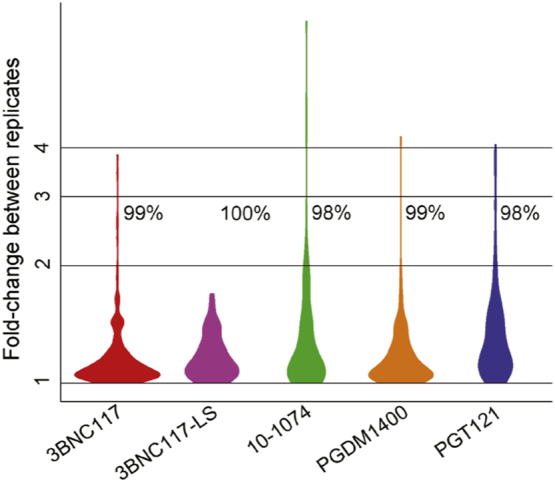
Determination of suitable titer step size and fold change threshold for Tier 3 characterization assay. Violin plots depicting the distribution of S/B fold changes observed between technical replicates among drug naïve subjects (*N* = 24–38). Inset values indicate the percent of replicate measurements demonstrating changes less than 3-fold.

Tier 3 ADA assays seek to define characteristics of confirmed ADA responses. Here, determination of response magnitude is accomplished by diluting sera samples and defining the extent to which a sample can be diluted while still exhibiting signal above the screening assay positivity cut point. An appropriate dilution step size and a corresponding fold-change in titer that is likely to be of biological rather than technical origin was defined using assay precision data. Across the 99+ samples from treatment naïve subjects tested to establish the QCP, any sample that tested Tier 1 positive in any of the six repeats (*N* = 24–38) were included in Tier 3 analysis. The distribution of fold changes observed between each replicate and the median of the six replicates in the screening assay was calculated for each bnAb (Fig. 7). Approximately 98% of repeated measures exhibited variability less than two-fold. However, balancing this low variability with practical constraints as to the number of dilution points necessary to define the response titer, and a magnitude change that might reasonably be expected to have potential biological significance, a three-fold dilution step and titer magnitude change was selected. Here, based on assay precision, we expect that applying a three-fold dilution step, and requiring a titer change of at least three-fold to define an increase in titer will result in a false positive rate of less than 2%. This step size sets the limit of resolution in defining longitudinal changes among positive samples, and is used to define treatment-boosted responses for subjects that were ADA positive at baseline.

### 3.9 Application of the tiered approach to NHP samples

**Fig. 8:**
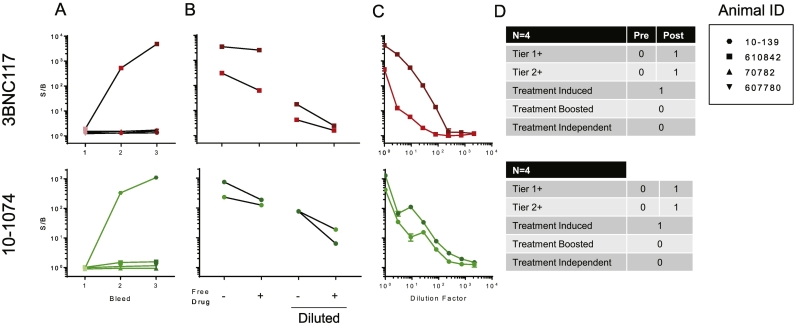
Implementation of the tiered approach to defining ADA responses among NHP administered bnAbs and sampled longitudinally. A. Tier 1 ADA testing of serum samples from NHPs that received 3BNC117 (top, red) and/or 10–1074 (bottom, green). Individual animals are connected by a line and have the same symbol. Timepoints are colored in different shades. B. Tier 2 ADA testing at either the standard serum dilution factor (left) or a more dilute concentration of serum (right). C. Tier 3 testing of samples that were deemed specific in Tier 2. D. A summary of the number of animals that were Tier 1 or 2 positive broken out by whether the samples that were positive were from animals before (pre) or after (post) they received drug. The type of ADA (treatment-induced, treatment-boosted, or treatment-independent) are also identified for each animal deemed Tier 2 positive. (For interpretation of the references to color in this figure legend, the reader is referred to the web version of this article.)

Though the procedures and criteria for the tiered ADA approach described above were defined on the basis of responses observed in treatment naïve human sera samples, we nonetheless sought to evaluate their suitability to define ADA responses among nonhuman primates administered bnAbs. Samples from 10 to 1074 alone (*N* = 1), and 10–1074 in combination with 3BNC117 (*N* = 3) treated animals, assessed longitudinally, were evaluated in the screening assay (Fig. 8A). For 3BNC117 or 10–1074-infused animals, a single animal each developed a response at later timepoints that exceeded the cut point defined by human samples, and this response was present at both follow-up timepoints. Of note, the sole animal that received 10–1074 alone was selected for testing from a larger cohort because it was suspected of having a potential ADA based on observation of rapid 10–1074 clearance. For each of the samples defined as screening assay positive, the confirmatory Tier 2 assay was performed to determine whether the observed responses were specific (Fig. 8B). All four Tier 1 positive samples for 3BNC117 and 10–1074 were deemed specific using the inhibition threshold criteria derived from human samples. Lastly, Tier 2 positive samples were titrated (Fig. 8C). Based on the sample testing paradigm (Fig. 1), each sample was defined as screening assay positive or negative; those positive in the screening assay were further defined as having confirmed or non-confirmed ADA responses, and subjects in which one or more timepoints exhibited a confirmed ADA response were categorized as exhibiting treatment-induced, treatment-boosted, or treatment-independent ADA (Fig. 8D) based on their longitudinal profiles.

## 4 Discussion

The development of ADA is an expected potential consequence of the passive infusion of any biological product (Song et al., 2016), though much is still unknown about the impact of these reactions or their frequency. Furthermore, even less is known about how subjects will tolerate anti-HIV bnAbs given their somewhat unique characteristics such as long complementarity determining regions (CDRs), framework mutations, and posttranslational modifications of CDRs (Yu and Guan, 2014; McLellan et al., 2011). Given the uniqueness of anti-HIV bnAbs, it has been reassuring to observe minimal ADA reactivity for VRC01 or an Fc-modified form of VRC01 (VRC01-LS), which have the characteristically long CDRs, in Phase I clinical trials (Ledgerwood et al., 2015; Gaudinski et al., 2018). Neither subcutaneous nor intravenous administration of VRC01, even after multiple doses, resulted in an ADA response exceeding the highest pre-infusion measurement (Ledgerwood et al., 2015). While ADA responses against 3BNC117 and 10–1074 have been detected in drug recipients, none have been associated with adverse events thought to be related to infusion, or considered to result in changes in elimination kinetics (Cohen et al., 2019). Conversely, it has been recently observed that adeno-associated virus-vectored expression of PG9, which also possesses long CDRs, did induce ADAs among the majority of study participants (Priddy et al., 2019). These ADAs were more easily detected than expressed bnAb, and they were often neutralizing. This apparent diversity in prevalence and consequences of ADA responses points toward the value of ADA testing as bnAbs are evaluated in new subject populations. To this end, as clinical testing of passive infusions of anti-HIV bnAbs for the prevention and treatment of HIV is a relatively recent but rapidly growing effort, establishment of a standardized method to detect and characterize ADAs will not only save valuable time and resources, but if done correctly, may enable comparisons across different programs evaluating the same drug product.

In these studies, a number of best practices regarding ADA detection and characterization have been employed. First, electrochemiluminiscence bridging-based methods were found to be reliable, reproducible, and highly sensitive. Second, a tiered approach to enhance sensitivity and identification of ADA positive samples was employed in conjunction with follow-up assays to confirm specificity and the magnitude of an ADA response. Though clearly conservative, as approximately 25% of samples are deemed Tier 1 positive, a strict threshold for Tier 2 is anticipated to ensure limited false positives. Finally, accepted statistical analyses were used to determine positivity and specificity. Once these assays were developed, evaluation of samples from non-human primate studies enabled proof of concept testing and reinforced confidence that the testing scheme is working as intended and capable of detecting anti-drug antibodies.

In developing assays for the five drug products described herein, it is important to note that the same approach was easily adapted for each of the products, setting the expectation that as other products become available, the same schema would be equally applicable and capable of conforming to current FDA standards. For each drug product, sensitivity meeting or exceeding FDA recommendations was observed. The relatively poorer sensitivity of the screening assay for PGT121 ADA may have less to do with the assay design, which in theory is the most sensitive due to the use of almost neat serum, and more to do with the specific anti-idiotype antibody used to establish the sensitivity. In preliminary testing with an alternate anti-idiotype antibody, we saw sensitivity that was more consistent with the four other drug products tested (data not shown). As expected based on the assay platform, matrix effects were generally negligible across all five drug platforms, enabling data collection in up to 93% serum. Such high tolerance to matrix components could allow even higher sensitivity if needed. Finally, reproducibility and robustness were both well within FDA tolerances and consistent with well-established clinical assays.

Of interest and as has been previously reported (Song et al., 2016; Kubiak et al., 2016; Kumar et al., 2017), the development of this assay has revealed a seemingly high incidence of treatment-naive individuals nominally positive for ADA responses (typically 16–20%) in Tier 1, some of which were relatively high in magnitude. The exact nature of these “baseline positives” are undefined, as it is not known whether positive signals result from immunoglobulins or some other serum component(s). Many responses observed among drug naïve subjects were considered specific based on being effectively competed with excess free drug. These samples were not bridged by other drug products and were not competed by mismatched antibodies (data not shown). In our limited experience to date, subjects who have high baseline ADAs prior to receiving drug product are not more likely to have a treatment-boosted ADA response than subjects with no baseline ADA response are to have a treatment-induced response (Cohen et al., 2019), but additional data are required to more conclusively define the significance of pre-existing ADA responses.

False negatives remain difficult to identify, particularly when drug levels are high, such as within the first several weeks or months post infusion due to interference in the assay and limited sampling windows that may miss a short-lived ADA. While the screening assay is reasonably tolerant to free drug, alternative assays to enable ADA monitoring during the early stages post-infusion based on precipitation and dissociation of ADA complexes may enable reduction of false negatives and support more confident testing of samples at early timepoints when high levels of drug are present (Zhong et al., 2017). Implementation of ADA testing across products and trials will define the biological relevance and implications of observed ADAs, which may or may not impact HIV bnAb pharmacokinetics, pharmacodynamics, biodistribution, toxicity, or the likelihood of adverse events.

## 5 Conclusion

In conclusion, assays have been developed and qualified for the detection and characterization of ADAs in subjects receiving HIV bnAbs 3NC117, 3BNC117-LS, 10–1074, PGT121, and PGDM1400 as either prophylactic or therapeutic agents. These assays were evaluated for sensitivity, specificity, precision, robustness, and the impact of free drug in circulation according to the recommendations made by the FDA, and proof of concept for their utility and suitability was demonstrated using samples from pre-clinical NHP studies in which ADAs were raised in response to passive infusion.

## Ethics statement

Human samples not procured from commercial sources were collected by Rockefeller University under purview of their IRB. Animal samples were obtained from a study conducted by David Garber at the Centers for Disease Control and Prevention which provided Institutional Animal Care and Use Committee oversight.

## Funding

These studies were conducted with support from the Bill and Melinda Gates Foundation [OPP1146996].

## Declaration of Competing Interest

There are no conflicts of interest to declare.

## Appendix A Supplementary data

Supplementary Data Tables Image 1
